# Electrochemical generation of sulfur vacancies in the basal plane of MoS_2_ for hydrogen evolution

**DOI:** 10.1038/ncomms15113

**Published:** 2017-04-21

**Authors:** Charlie Tsai, Hong Li, Sangwook Park, Joonsuk Park, Hyun Soo Han, Jens K. Nørskov, Xiaolin Zheng, Frank Abild-Pedersen

**Affiliations:** 1 SUNCAT Center for Interface Science and Catalysis, Department of Chemical Engineering, Stanford University, 443 Via Ortega, Stanford, California 94305, USA; 2 SUNCAT Center for Interface Science and Catalysis, SLAC National Accelerator Laboratory, 2575 Sand Hill Road, Menlo Park, California 94305, USA; 3 Department of Mechanical Engineering, Stanford University, 440 Escondido Mall, Stanford, California 94305, USA; 4 School of Mechanical and Aerospace Engineering, Nanyang Technological University, Singapore 639798, Singapore; 5 Department of Material Science & Engineering, Stanford University, 440 Escondido Mall, Stanford, California 94305, USA

## Abstract

Recently, sulfur (S)-vacancies created on the basal plane of 2*H*-molybdenum disulfide (MoS_2_) using argon plasma exposure exhibited higher intrinsic activity for the electrochemical hydrogen evolution reaction than the edge sites and metallic 1*T*-phase of MoS_2_ catalysts. However, a more industrially viable alternative to the argon plasma desulfurization process is needed. In this work, we introduce a scalable route towards generating S-vacancies on the MoS_2_ basal plane using electrochemical desulfurization. Even though sulfur atoms on the basal plane are known to be stable and inert, we find that they can be electrochemically reduced under accessible applied potentials. This can be done on various 2*H*-MoS_2_ nanostructures. By changing the applied desulfurization potential, the extent of desulfurization and the resulting activity can be varied. The resulting active sites are stable under extended desulfurization durations and show consistent HER activity.

## Abstract (web summary)

In order to fully utilize sulfur vacancies in MoS_2_ catalysts for industrial applications, a facile and general route for making sulfur vacancies in MoS_2_ is needed. Here, the authors introduce a scalable route towards generating sulfur vacancies on the MoS_2_ basal plane using electrochemical desulfurization.

Molybdenum disulfide (MoS_2_)-based catalysts for the electrochemical (EC) hydrogen evolution reaction (HER) have attracted intensified research in recent years due to their earth abundance and potential to become highly active alternatives to platinum catalysts. Ever since the edge sites of 2*H*-MoS_2_ were theoretically predicted^1^ and experimentally verified^2^,^3^ to be the active sites for hydrogen evolution, subsequent efforts have largely focused on maximally exposing such edge sites through nano-structuring techniques^3^,^4^,^5^. The basal plane, which usually constitutes the bulk of the material, has only recently been considered as a possible source of active sites, with the 1*T*-phase^6^,^7^,^8^ being one of the first examples. Very recently, we demonstrated that active sites could be directly created on the basal plane of common 2*H*-MoS_2_ materials by generating sulfur (S)-vacancies, whose intrinsic activity can be optimized by fine tuning the S-vacancy concentration and introducing elastic tensile strain^9^. At the S-vacancy sites, the under-coordinated Mo atoms introduce gap states that allow for favourable hydrogen binding, leading to the highest per-site turnover frequency (TOF) reported for any MoS_2_-based catalyst for HER. Though MoS_2_ catalysts are increasingly being considered for various electro-catalytic processes, the potential roles of these newly discovered S-vacancy sites are still unknown. In addition, the S-vacancies in the basal plane have so far only been generated using controlled argon (Ar) plasma exposure and H_2_ annealing^10^. This approach is only effective for flat MoS_2_ catalysts due to the Ar plasma’s directionality and is thus unsuitable for large-scale synthesis^11^. As such, the possibility of creating S-vacancies in other MoS_2_-based materials has not yet been demonstrated. To fully utilize S-vacancies in MoS_2_ catalysts for industrial applications, a facile, general and scalable route for generating S-vacancies in MoS_2_ of any morphology is needed.

One possibility for generating S-vacancies is to remove sulfur atoms from the basal plane electrochemically. This idea involves reducing the sulfur atoms in the basal plane of 2*H*-MoS_2_ to form hydrogen sulfide (H_2_S) gas via a desulfurizing ‘activation’ cycle, similar to the oxygen vacancies generated in metal oxides under applied cathodic potentials^12^.

Herein, we present an EC process for generating S-vacancies in monolayer as well as polycrystalline multilayer MoS_2_ supported on various electrodes. We use density functional theory (DFT) calculations to show that the formation of S-vacancies in the basal plane is expected to be thermodynamically favourable at a sufficiently reducing potential. Sulfur atoms in the basal plane are hydrogenated and then removed as H_2_S gas to form the S-vacancies. The concentration of S-vacancies can be varied by changing the applied desulfurization voltage. These predictions are experimentally verified on a well-defined model system of continuous MoS_2_ monolayers supported on gold (Au) (the same system considered previously^9^), thus showing that electrochemically generated S-vacancies are comparable to Ar plasma generated ones. We further demonstrate the generality of the EC approach by electrochemically generating S-vacancies on multilayered MoS_2_ supported on flat carbon rods and porous carbon foam, leading to significantly improved HER activity in both cases. Finally, using MoS_2_ supported on carbon foam, we show that the HER activity is stable under extended desulfurization durations as well as operating durations and that the concentration of S-vacancies and activity can be varied using the applied potential.

## Results

### Theoretical predictions

**Figure 1:**
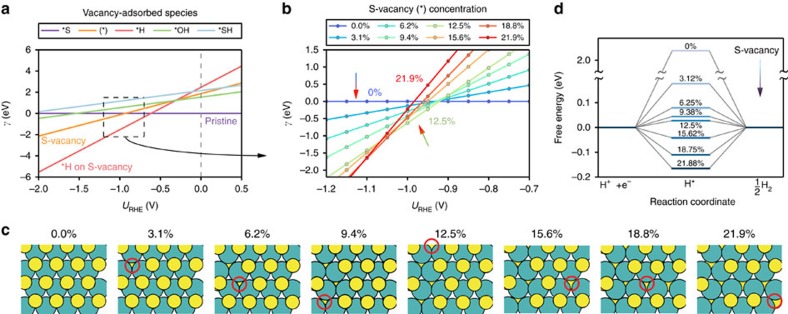
Theoretical predictions. (**a**) Surface energy per unit cell for 2*H*-MoS_2_ as a function of applied potential for the basal plane of 2*H*-MoS_2_ with different adsorbate species at a fixed sulfur vacancy (3.1%). The surface energies of various species in the S-vacancy are shown. (*) Refers to a S-vacancy and the adsorbed species in the S-vacancy are denoted by *prefix. *S refers to sulfur adsorbed in the vacancy, that is, a pristine basal plane without S-vacancies. (**b**) Surface energy per unit cell for a range of S-vacancy concentrations, without any adsorbates. The concentration of S-vacancies varies from 0 to 21.9% within the narrow range of −1.0 V to −1.1 V. In all cases, the surface energies are taken relative to a pristine basal plane (*γ*=0 eV). (**c**) When the S-vacancy sites are generated in succession, S-vacancies are most stable when formed next to an existing S-vacancy. (**d**) Free energy diagram for the HER on S-vacancy sites. The results represent hydrogen adsorption on the most stable site within the computational cell.

Using DFT calculations, we investigated the conditions where S-vacancies in the 2*H*-MoS_2_ basal planes become thermodynamically favoured in an EC environment. Using a descriptor-based analysis with the hydrogen adsorption free energy^9^, we showed previously that edge vacancies are not expected to increase HER activity. Hence, we focus on the basal plane in this work. The surface free energies per unit cell (relative to pristine MoS_2_) as a function of the applied potential were determined for pristine MoS_2_, MoS_2_ with S-vacancies (3.1% surface vacancies) and MoS_2_ with various species adsorbed on the S-vacancy (Fig. 1a). The exact values of the surface energies depend on a reference pressure of H_2_S gas, which we have chosen as 10^–8^ bar according to standard corrosion resistance^13^. Although the energies of all surfaces involving a vacancy will be lower if a smaller pressure is chosen, the qualitative trends and relative energies between the different surfaces are independent of the reference.

At around −1.0 V versus RHE, the surface with S-vacancies (*) becomes stable compared to the pristine basal plane (*S). Hydrogen adsorption onto the S-vacancy site (*H) becomes more thermodynamically favoured compared to either the re-adsorption of sulfur (*S) below −0.3 V versus RHE (Fig. 1a) or *OH poisoning. This indicates that generated S-vacancies will be immediately occupied by hydrogen and that the removed H_2_S gas is unlikely to return. Hence, only H atoms are expected to adsorb in the generated S-vacancies throughout the desulfurization process and even after the applied potential is turned off. It should then be possible to permanently activate the MoS_2_ basal plane in a desulfurizing step.

We further compare the surface energies per unit cell (relative to pristine MoS_2_) of an empty S-vacancy as a function of the applied potential for a wide range of S-vacancy (*S) coverages (Fig. 1b shows a zoomed-in region of the applied potential). Larger amounts of vacancies are stabilized at more negative potentials, indicating that the applied potential can be used to vary the concentration of S-vacancies. The formation of 3.1% S-vacancies on the surface (γ≤0 eV) occurs around −1.0 V versus RHE, which is similar to the surface energies for both 6.2 and 9.4% S-vacancies but higher than that of 12.5% S-vacancies. This is because vacancies tend to form in clusters to stabilize the system (Fig. 1c, see Supplementary Table 1, Supplementary Fig. 1, Supplementary Note 2 and a transmission electron microscopy (TEM) image in Supplementary Fig. 2 and Supplementary Note 3 for further details of this behaviour), and clustered vacancies have lower energies than evenly dispersed ones. Once the first S-vacancy is formed, successive S-vacancies are more readily made in their vicinity. They first follow a zigzag pattern and then branch out once a continuous repeating zigzag (within our computational cell) is formed. The lowest energy of 12.5% corresponds to a periodically repeating and continuous zigzag of S-vacancies in the computational cell. We note that there could be intermediate coverages of S-vacancies following the zigzag configuration that are not representable by the until cell size in this study.

Beyond 12.5% vacancies, however, the energetic cost of forming a vacancy outweighs the stabilization from forming clusters of vacancies. Although it appears that either a 12.5, 15.6 or 21.9% coverage of S-vacancies will be most stable at potentials lower than −1.0 V versus RHE, many of them are similar in energy. There could also be additional kinetic barriers towards forming the S-vacancies that are not yet considered here. Regardless, the entire range of S-vacancy coverages considered herein becomes thermodynamically accessible below −1.0 V versus RHE.

We showed recently that the optimal hydrogen adsorption free energy Δ*G*_H_=0 eV for HER occurs for an S-vacancy concentration that is between 12.5 and 15.62% of the surface atoms. Although our previous results were performed on MoS_2_ with evenly spread out S-vacancies, we find that the Δ*G*_H_ and associated trends are nearly the same on MoS_2_ with clustered vacancies (Fig. 1d). The values of Δ*G*_H_ are well within 0.1 eV of each other. The 12.5 and 15.62% concentrations are lowest in free energy at potentials from −0.97 to −1.15 V versus RHE (Fig. 1b). The values of Δ*G*_H_ for all other S-vacancy concentrations are also within ±0.1 eV and correspond to highly active sites for HER.

**Figure 2:**
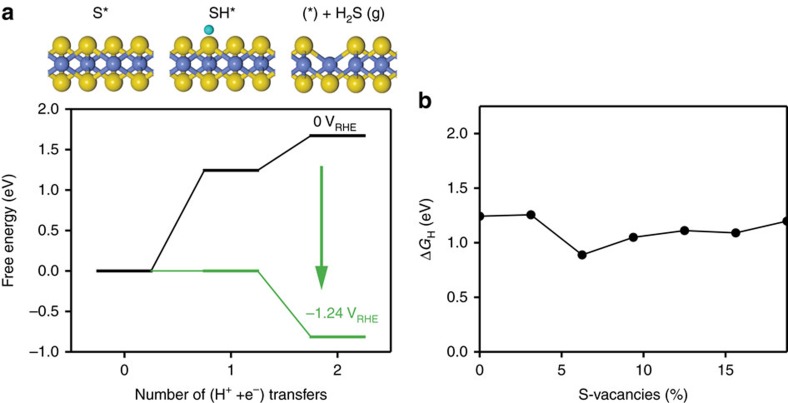
The EC desulfurization process. (**a**) Free energy diagram for the protonation and removal of S. The green line indicates the free energy pathway at the applied potential required to make all paths exergonic. An illustration of the EC desulfurization process corresponding to the steps is shown above the plot. (**b**) Hydrogen adsorption free energy (Δ*G*_H_) onto a sulfur atom on the basal plane for each concentration of S-vacancies.

The surface energies only reflect the overall thermodynamic process and do not take into account the intermediate steps, which could have larger energetic requirements. However, it is useful for comparing the relative stabilities of the different desulfurization extents. To verify whether the surface energies adequately reflect the trends, the full reaction pathway needs to be determined as well. The desulfurization process is expected to occur via the proton and electron transfer to the sulfur atoms in the basal plane: *S+2(H^+^+*e*^−^)→*+H_2_S_(g)_, where the hydrogenated sulfur atom (*SH) is removed as H_2_S gas, leaving behind a S-vacancy (*) (Fig. 2a). Here * denotes a S-vacancy and the * prefix indicates a species adsorbed on the S-vacancy. The pristine basal plane is thus referred to as *S. This process can occur through the two proton–electron transfer steps as follows:


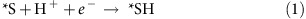



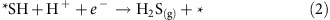


Using the computational hydrogen electrode, the chemical potential of the proton–electron pair can be calculated relative to that of hydrogen gas, allowing for the potential-dependent free energy to be determined^14^,^15^ (see Methods section). The reaction steps are shown in the free energy diagram and the schematic in Fig. 2a. At 0 V versus RHE, the first protonation to form *SH (equation 1) is uphill by 1.24 eV and hydrogen adsorbs extremely weakly, in agreement with the observation that the pristine basal plane of MoS_2_ is inert towards hydrogen evolution at moderate potentials (solvation effects have been included in the calculations using one layer of explicit charged solvent^16^, where Δ*G*_H_ was found −0.65 eV lower compared to previous calculations in vacuum^17^,^18^). Adsorption of the second proton to form gaseous H_2_S (equation 2) is 0.42 eV uphill from the first step. Nevertheless, both steps become exergonic at a potential of −1.24 V versus RHE. This is a ∼0.24 V higher cathodic potential than the required potential determined by the equilibrium surface energy diagrams (Fig. 1b), indicating that the desulfurization reaction needs to overcome an additional 0.24 eV compared to the results from the surface energy diagrams. However, the Δ*G*_H_ required for reaction step in equation (1) is approximately the same for all S-vacancy concentrations studied herein (Fig. 2b), which indicates that the surface free energies of the S-vacancies (Fig. 1b) will determine which S-vacancy concentration is most stable. The results in Fig. 1 should thus sufficiently describe those trends qualitatively.

### Experimental verification

**Figure 3:**
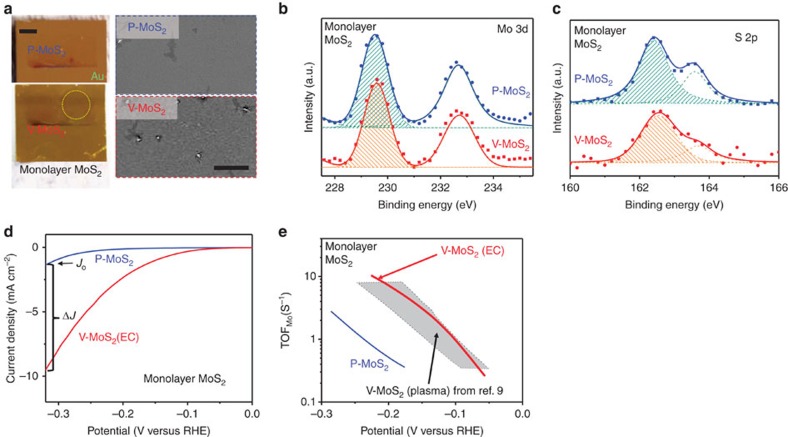
**Characterizations and catalytic activity of monolayer MoS**_**2**_. (**a**) Optical (left panel; scale bar, 2 mm) and SEM images (right panel; scale bar, 20 μm) of monolayer MoS_2_ film before (P-MoS_2_, upper panel) and after (V-MoS_2_, lower panel) desulfurization. (**b**) XPS Mo 3d and (**c**) S 2p peaks of pristine MoS_2_ (P-MoS_2_, upper curves) and MoS_2_ with S-vacancies (V-MoS_2_, lower curves). The filled symbols are measured data, and the solid lines are the parametric fits. The dashed lines with enclosed shaded areas label the major peak used to characterize the S:Mo atomic ratio. (**d**) LSV of monolayer MoS_2_ before and after desulfurization, respectively. The current density increment is defined as Δ*J*/*J*_0_, where *J*_0_ is the current density at −0.32 V versus RHE before desulfurization. (**e**) TOF per surface Mo atom (TOF_Mo_) as a function of applied potential for MoS_2_ monolayers before and after desulfurization. The results from previous work where Ar plasma was employed for desulfurization are also shown for comparison (shaded area). All the current densities are defined based on the total electrode surface area.

To experimentally verify our theoretical predictions, we tested the EC process for generating S-vacancies in monolayer and multilayer MoS_2_ catalysts supported on various electrodes. We first synthesized continuous 2*H*-MoS_2_ monolayers with large single-crystal domains (>100 μm) supported on a Au substrate (see Method section for details). This well-defined model system maximizes the exposure of basal plane sites and minimizes the presence of edge sites, which enables us to unambiguously identify the effects of the desulfurization process on the HER activity of the basal planes of 2*H*-MoS_2_. This also allows us to draw direct comparisons with our recent work, where S-vacancies were generated using Ar plasma treatment on the exact same model system. Since S-vacancies are expected to become stable starting at –1.0 V versus RHE (Fig. 1a,b), the desulfurization process was performed by decreasing the potential to −1.0 V versus RHE using linear sweep voltammetry (LSV) with a sweeping speed of 50 mV s^–1^. After the desulfurization process, the desulfurized sample with vacancies (V-MoS_2_, Fig. 3a) shows observable morphological change (scanning electron microscopy (SEM) image) compared to the pristine as-synthesized catalyst (P-MoS_2_, Fig. 3a) suggesting the formation of defects.

To further confirm the selective removal of S atoms by the EC desulfurization process, we compared the S:Mo atomic ratio between P-MoS_2_ and V-MoS_2_ using X-ray photoelectron spectroscopy (XPS). The Mo 3d peaks (Fig. 3b) are similar for P-MoS_2_ and V-MoS_2_, suggesting that the Mo atoms are not significantly affected. However, the S 2p peaks for V-MoS_2_ are much weaker than those of P-MoS_2_ (Fig. 3c), confirming the removal of S atoms during the desulfurization process. For these particular XPS spectra, the S:Mo peak area ratio for V-MoS_2_ is about 25% smaller than of P-MoS_2_, corresponding to ∼25% S-vacancies. We used the major peaks (marked as shaded areas in Fig. 3b,c) to calculate the atomic ratio of S:Mo while that of the as-grown MoS_2_ is normalized to 2.0. We note that the S-vacancy percentage was not spatially uniform across the sample surface, which could be due to the clustering of S-vacancies observed in the DFT results and TEM image (Supplementary Fig. 2).

We further compared the HER catalytic activity of V-MoS_2_ (EC) with P-MoS_2_. As shown in Fig. 3d, the current density of V-MoS_2_ (EC) is considerably higher than that of P-MoS_2_. Any activity from P-MoS_2_ likely comes from the minority of exposed edge sites in the continuous monolayers or intrinsic defects in the catalyst, which would create edges or basal plane vacancies. To quantify the improvement, we define a normalized current density increment Δ*J/J*_0_, where *J*_0_ is the current density of P-MoS_2_ and Δ*J* is the current density increase at −0.32 V versus RHE (Fig. 3d). We tested six monolayer MoS_2_ samples with an average S-vacancy concentration about (15±8)%, and their Δ*J/J*_0_ values range (438±159)%, confirming the generation of catalytically active S-vacancies using the EC process. We then calculated the TOF normalized to the number of surface Mo atoms (TOF_Mo_) to compare the intrinsic HER activities for the S-vacancy sites. As shown in Fig. 3e, the TOF_Mo_ for V-MoS_2_ (EC) is more than an order of magnitude higher than that of P-MoS_2_ in certain potential ranges. Importantly, the TOF_Mo_ for V-MoS_2_ (EC) is comparable to the previously reported V-MoS_2_ (Ar plasma treatment^9^) with the same range of S-vacancy concentrations (grey colour area in Fig. 3e). The quantitative comparison clearly shows that the EC desulfurization process is as effective as the Ar plasma treatment^9^ for generating highly active S-vacancy sites. Finally, it should be noted that S-vacancies are not only active for HER but also improves the electron injection from the MoS_2_ to the substrate^19^. Both factors contribute to the observed high-intrinsic HER activity of the S-vacancy.

**Figure 4:**
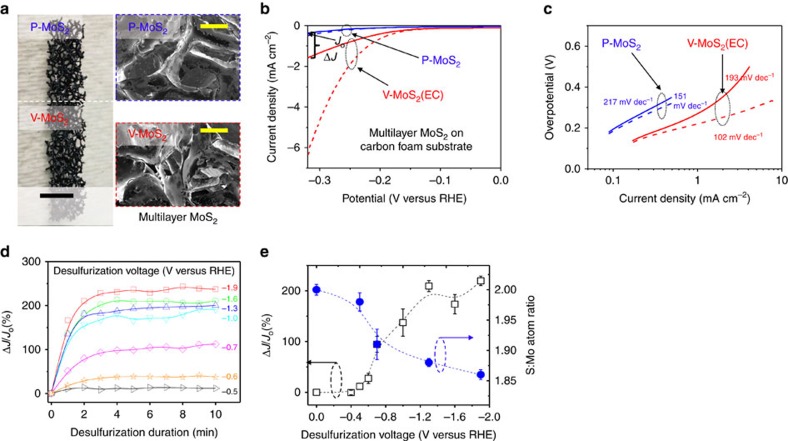
**HER activity before and after desulfurization of polycrystalline multilayer 2*****H*****-MoS**_**2**_. (**a**) Optical (left panel; scale bar, 0.5 cm) and SEM images (right panel; scale bar, 0.5 mm) of multilayer MoS_2_ film on carbon foam before (P-MoS_2_, upper panel) and after (V-MoS_2_, lower panel) desulfurization at −1.3 V versus RHE for 60 s. (**b**) LSV of multilayer MoS_2_ on carbon foam before and after desulfurization, respectively. All the current densities are defined based on the total electrode surface area. The current density increment is defined as Δ*J*/*J*_0_, where *J*_0_ is the current density at −0.32 V versus RHE before desulfurization. The J–V curves after iR correction are plotted using dashed curves for comparison. (**c**) The corresponding Tafel plot of the curves in **b**. The calculated Tafel slopes are labelled for each curve. (**d**) Current density increment versus desulfurization duration at various desulfurization voltages from −0.5 to −1.9 V versus RHE. (**e**) Current density increment (left, *y* axis) and S:Mo atom ratio (right, *y* axis) versus desulfurization voltage at fixed desulfurization duration of 10 min. The error bars represent the s.d.'s.

The EC desulfurization process should be a general procedure for generating S-vacancies on any MoS_2_ electrodes since it simply reduces S atoms on the surface. It thus acts as a post-synthesis activation procedure. To verify this, we applied the desulfurization process to various 2*H*-MoS_2_ systems including polycrystalline 2*H*-MoS_2_ grown on commercial carbon rods and foams^20^ (Fig. 4a). The desulfurization process was performed by applying a constant voltage using chronoamperometry with a 60 s interval unless specified otherwise. These supported polycrystalline multilayer MoS_2_ materials differ from monolayer MoS_2_ (Fig. 3) in both surface area and electrode morphology. After the desulfurization process, the polycrystalline multilayer MoS_2_ supported on carbon foam appears the same through optical and SEM inspection (Fig. 4a), but their HER catalytic activity shows a pronounced improvement with a Δ*J*/*J*_0_ of about three-fold (333%) (Fig. 4b). The activity enhancement caused by desulfurization is even more obvious if we compare the J–V curves after iR correction (dashed curves). Comparing the iR-corrected curve of P-MoS_2_ (blue dashed curve) and V-MoS_2_ (red dashed curve), there is an ∼12 times current density increment (at −0.32 V versus RHE) due to the desulfurization (corresponding Tafel plot shown in Fig. 4c). The Tafel slopes are greatly decreased due to desulfurization in both cases for before (from 217 to 193 mV dec^–1^) and after iR correction (151 to 102 mV dec^–1^). These comparisons suggest that the HER kinetics of the desulfurized sample is much faster than that of the pristine sample^21^.

Having demonstrated the generality of the EC S-vacancy generation, we then investigated how the HER activity can be varied w.r.t. the desulfurization duration and the desulfurization voltage. For this study, we used the polycrystalline multilayer MoS_2_ supported on carbon foam because they are more durable than the MoS_2_ monolayers, and have better performance than those on carbon rods. An 8 h long stability test was performed, where the current was stable for the full duration (Supplementary Fig. 4 and Supplementary Note 5). As shown in Fig. 4d, the desulfurization process was carried out at different cathodic potentials. Under each potential, the desulfurization process was carried out in an interval up to 10 min. Overall, the HER activity of MoS_2_ multilayers improves with increasing desulfurization voltage. For each desulfurization voltage, the HER activity of MoS_2_ multilayers also first increases with increasing desulfurization duration and then levels off. This is likely due to the competition between the desulfurization reaction and HER. As more S-vacancies are generated, more sites could become available for HER, which inhibits further desulfurization and leads to a steady-state coverage of S-vacancies. There is thus minimal risk in fully desulfurizing the MoS_2_ into Mo metal.

We further plotted the steady-state Δ*J/J*_0_ (desulfurization with 10 min interval in Fig. 4d) as a function of the applied desulfurization voltage (Fig. 4e, left *y* axis). The corresponding S:Mo atomic ratios for those conditions were measured using inductively coupled plasma optical emission spectroscopy (ICP-OES) (Fig. 4e, right *y* axis). First, the desulfurization process has an onset potential of about −0.6 V versus RHE. According to our theoretical predictions (Fig. 1a), this is where hydrogen begins to adsorb onto existing vacancy sites. Second, the desulfurization is most effective between −0.6 and −1.2 V versus RHE as indicated by the sharp decrease of S:Mo atomic ratio and increase of Δ*J/J*_0_. In line with our predictions, this is the region where a vacancy-rich surface and a pristine surface are predicted to have comparable equilibrium surface energies (Fig. 1). Finally, the desulfurization effect levels off beyond −1.3 V versus RHE. The levelling off of the current density increment is predicted by our theoretical calculation in that successive S-vacancies generation becomes increasingly difficult at higher S-vacancy concentrations (Fig. 1b). The desulfurization voltage and the desulfurization time are thus effective knobs for tuning the final activity of the vacancy-rich MoS_2_ catalysts.

**Figure 5:**
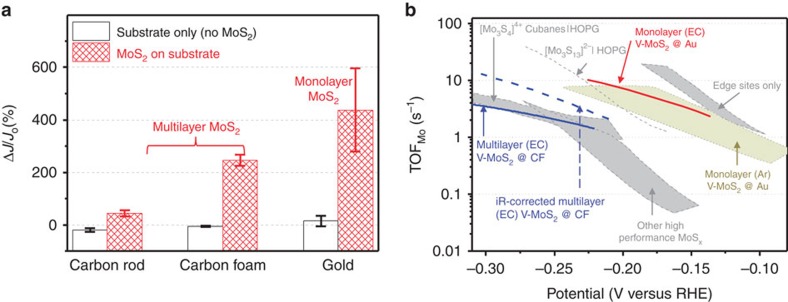
**Summary of the HER activity of electrochemically desulfurized MoS**_**2**_. (**a**) Summary of the desulfurization effect on the enhancement of HER activity for multilayer MoS_2_ on carbon rods and carbon foam substrates, and monolayer MoS_2_ on Au substrates. The unfilled bars illustrate the effect of electrochemically desulfurization process on the substrates themselves and the effect is negligible. The error bars represent the s.d.’s among the samples. (**b**) Comparison of turnover frequencies TOF_Mo_ per surface Mo atom between our samples and state-of-the-art MoS_*x*_ electrocatalysts. Various state-of-the-art MoS_*x*_ catalysts from ref. 24 are shown with non-bold labels. The light-green-coloured area is for Ar-desulfurized monolayer MoS_2_ from ref. 9. The red solid curve is for electrochemically desulfurized monolayer MoS_2_ on an Au substrate. The blue solid curve is for electrochemically desulfurized multilayer MoS_2_ on carbon foam substrate without iR correction. The blue dashed curve is from electrochemically desulfurized multilayer MoS_2_ on a carbon foam substrate with iR correction.

We also conducted a control experiment by applying the desulfurization process to the support only (without MoS_2_). Figure 5a summarizes the effect of the EC desulfurization process on all samples (See Supplementary Fig. 6 for detailed sample statistics). First, the support substrate (carbon rod, carbon foam and Au) did not show any observable current density enhancement (Fig. 5a, unfilled bars), indicating that the desulfurized MoS_2_ catalyst is responsible for the enhanced HER activity. Second, the EC desulfurization process improves the HER activity of MoS_2_ monolayers the most, which is due to the predominance of basal plane sites in the continuous pristine monolayers and absence of Ohmic loss due to interlayer tunnelling^22^. Third, the enhancement effect is smaller for the MoS_2_ multilayers supported on carbon. This is partly due to the polycrystalline nature of the multilayers (grain size <100 nm), which will have a relatively higher ratio of edge sites to basal plane sites than that of a perfect monolayer. Hence, the edge sites contribute to the measured HER activity even after desulfurization. Even in this case, the desulfurization process can triple the current density, confirming a significant contribution from the activated basal plane sites.

### Comparisons to state-of-the-art catalysts

It should be noted that the current density and Tafel slopes of the desulfurized MoS_2_ are lower than some of the best MoS_2_ electrodes reported in the literature^6^,^7^,^23^. This is partially because our current density was normalized to the total electrode area rather than the projected area, and the morphology of the MoS_2_ catalysts have not yet been optimized in this work. The current densities and Tafel slopes are also greatly affected by the loading of the catalyst. It is important to plot the intrinsic activity, in term of TOF per surface Mo atom, to make a fair comparison between different catalysts and understand the effect of EC desulfurization independent of catalyst loading. Figure 5b shows the TOF per surface Mo atom (TOF_Mo_) versus applied potential of our samples, together with those MoS_*x*_-based HER electrocatalysts considered state of the art. The grey curves represent these MoS_*x*_ electrocatalysts, including edge sites, amorphous cluster, nanowire, mesoporous film and so on, summarized in ref. 24. The upper right light-green-coloured area shows the TOF_Mo_ range of Ar-desulfurized monolayer MoS_2_ (ref. 9). The red and blue solid curves represent the EC desulfurized monolayer MoS_2_ on Au substrates and multilayer MoS_2_ on carbon foam substrates without iR correction, respectively. And the blue dashed curve represents the EC desulfurized multilayer MoS_2_ on carbon foam with iR correction. One can see that (1) the TOF_Mo_ of our desulfurized monolayer MoS_2_ (red solid curve) is close to the highest TOF_Mo_; and (2) the TOF_Mo_ values of our desulfurized multilayer MoS_2_ catalysts are comparable and slightly higher than most state-of-the-art MoS_*x*_. They are only lower than edge sites of monolayers (which disregards non-edge sites in the normalization) and amorphous [Mo_3_S_13_]^−2^ supported on HOPG (see Supplementary Table 2 and Supplementary Fig. 6 for a quantitative comparison of potential). Our results not only illustrate a general method of EC desulfurization that can be applied to different catalysts, but display high-intrinsic activity in all cases, comparing favourably to the most active sites reported so far. Since our sites are generated from the inert basal plane, our active sites should in principle, work in conjunction with other active sites such as the edge sites.

Although we have focused on HER, the simplest EC reaction, the exact same process can be carried out to generate sulfur vacancies for any other process of interest. MoS_2_-type materials are increasingly being considered for various EC processes such as the electro-catalytic reduction of CO_2_ (refs 25, 26). There the operating potentials are much closer to the desulfurization potentials, so the possibility of vacancy formation has significant implications. The surface functionalization of MoS_2_ in field-effect transistors^27^ is another very recent example outside of catalysis where surface vacancies have been exploited. Finally, the formation of surface vacancies simply involves the EC reduction of the chalcogenide on the surface, so similar surface vacancies could in principle be created in other sulfides, selenides, phosphides, oxides and nitrides as well. Our work demonstrates an effective proof of concept for accomplishing this.

## Discussion

In summary, we have theoretically devised and then experimentally validated a facile and scalable method for electrochemically generating catalytically active S-vacancy sites on *2H-*MoS_2_ under ambient conditions. Once the sulfur atoms are electrochemically reduced, the catalyst is permanently activated. We have demonstrated the generality of this method by applying it to various systems of MoS_2_ catalysts, which all show significant improvement in HER activity. The sulfur removal and hence activity increment can be further tuned by varying the applied desulfurization voltage and duration. Although we demonstrate a high per-site TOF, it is worth noting that our samples have limited total activity, which must also be optimized before these catalysts can be considered true viable alternatives to the state-of-the-art. This can be done using the various nano-structuring techniques mentioned earlier, and is the subject of ongoing work. Since the 2*H*-MoS_2_ basal plane constitutes the bulk of industrial MoS_2_ catalysts and is present in almost all MoS_2_ catalysts, our approach can be used directly to further enhance the HER activity on all of those catalysts.

## Methods

### Theoretical calculations

All calculations in this work were performed using plane-wave DFT employing periodic boundary conditions as implemented in the Quantum ESPRESSO code. The Bayesian error estimation exchange-correlation functional with van der Waals interactions^28^ was used. This functional has been optimized for chemisorption energies as well as van der Waals interactions. The lattice parameters of MoS_2_ were determined to be *a*=3.19 Å and *c*=13.05 Å, in close agreement with experimentally measured values of *a*=3.162 Å and *c*=12.29 Å (ref. 29). A plane-wave cutoff of 500 eV and a density cutoff of 5,000 eV were used, and a Monkhorst–Pack 2 × 2 × 1 *k*-point grid was used to sample the Brillouin zone. Periodic boundary conditions were used in all directions and 11 Å of vacuum was used in the *z* direction to separate the slabs, which are 4 Mo atoms × 4 Mo atoms in size. For the structural relaxation, a convergence criterion of 0.05 eV Å^–1^ was used for the maximum force. The reaction free energies, Δ*G*^0^, are determined at standard conditions and a potential of *U*=0 V as


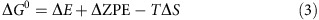


where Δ*E* is the reaction energy, ΔZPE is the difference in zero point energies and Δ*S* is the change in entropy for the reaction step. Additionally, the potential dependence of hydrogen adsorption is including using the computational hydrogen electrode model^14^,^15^, where


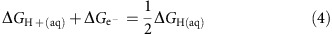


at a potential of *U*=0 V versus RHE. The effect of an applied potential can then be included by shifting the free energy using Δ*G*=Δ*G*^0^+*eU*. The results in the free energy diagram shown in Fig. 2 were determined using this approach. Although reaction barriers were not calculated, they are known to scale with reaction energies^30^, so our results should still be sufficient for illustrating the relevant trends. Further information about the computational set-up is described in Supplementary Note 1.

### Fabrication of continuous 2*H*-MoS_2_ monolayers on Au electrode

A customized tube furnace system was employed to grow continuous 2*H*-MoS_2_ monolayers. As-purchased molybdenum trioxide (MoO_3_) and sulfur (S) powder were loaded in alumina boats separately. The alumina boat containing MoO_3_ was placed in the centre of the tube furnace where the temperature was set at 750 °C during the growth, and the alumina boat containing S was placed upstream where the temperature was about 200 °C during the growth. Argon gas was used as the carrier gas to bring S vapour to the centre of the tube furnace at atmospheric pressure. A SiO_2_/Si wafer suspended on the alumina boat was used to as the growth substrate for the MoS_2_ monolayers. The as-grown MoS_2_ monolayers are almost continuous in an area larger than 3 mm in diameter (the size of our EC compression cell) for the EC measurements. A continuous MoS_2_ monolayer with high coverage and large flake size was selected and transferred onto Au (80 nm)/titanium (10 nm)-coated SiO_2_/Si wafer, and cleaned thoroughly in hot acetone (56 °C for 10 min) and chloroform (60 °C for 1 h) sequentially. As both solvents vaporize quickly when heated up, the beaker was partially covered to reduce the loss of solvent.

### Fabrication of polycrystalline 2*H*-MoS_2_ on carbon electrodes

Polycrystalline multilayer 2*H*-MoS_2_ was grown on commercial carbon rods and carbon foams through thermolysis of ammonium thiomolybdates [(NH_4_)_2_MoS_4_]. Commercial (NH_4_)_2_MoS_4_ was dissolved in dimethylformamide using a concentration of 1.25 wt%. The solution was stirred and sonicated to completely dissolve (NH_4_)_2_MoS_4_ particles. Then, the cleaned carbon rod and foam were dipped in the solution, and then dried in air at 50 °C. The carbon electrodes with coated (NH_4_)_2_MoS_4_ were heated in a tube furnace in the presence of S vapour with argon as the carrier gas at 400 °C and 1 atm for 1 h. During the heating process, (NH_4_)_2_MoS_4_ decomposed and formed polycrystalline MoS_2_ (see Supplementary Fig. 3 and Supplementary Note 4 for Raman spectra^31^). The control samples that contain the carbon substrates only (Fig. 4d) were fabricated using the same sulfurization process.

### XPS measurements

XPS characterizations were conducted in UHV (5 × 10^−10^ Torr) with excitation by Al (ka) radiation. The S:Mo atom ratios were estimated from the XPS peak area ratio of S 2p to that of Mo 3d states. The XPS peak area ratio of S 2p to Mo 3d states for as-grown pristine MoS_2_ was set as 2.0 to normalize other samples.

### ICP-OES measurements

ICP-OES was carried out using a Thermo scientific ICAP 6300 Duo View Spectrometer. A commercial high-quality standard solution of Mo and S elements with 10 ppm concentration in 2% nitric acid was used. As a blank solution 2.8% nitric acid was used and mixed with the standard solution to make 2.4% nitric acid of the quality control standard solution. MoS_2_ samples were dissolved in 69% concentrated nitric acid solvent for 18 h. Then the impurity particles of the sample solution were removed by centrifugation at a speed of 4,000 rpm for 15 min. Finally, a clear solution was taken and diluted to 8% nitric acid solution and injected into ICP-OES to get the elemental ratio of Mo and S.

### EC measurements

A Gamry 1000 potentiostat was employed for the EC measurements in this work. A three-electrode EC cell was used with 0.5 M sulfuric acid (H_2_SO_4_) as the electrolyte (the desulfurization process was performed in the same electrolyte). The working electrode was MoS_2_, and the counter electrode was a Pt wire. The reference electrode was a commercial Ag|AgCl electrode. The desulfurization of monolayer MoS_2_ samples (Fig. 3) was performed using LSV with a sweeping speed of 50 mV s^–1^. The desulfurization for the multilayer MoS_2_ samples (Fig. 4) was performed using chronoamperometry with a 60 s time interval unless specified otherwise. The stability of desulfurized polycrystalline multilayer MoS_2_ was tested at constant potential of −0.32 V versus RHE (Supplementary Fig. 4 and Supplementary Note 5).

### Data availability

The data that support the findings of this study are available from the corresponding author on reasonable request.

## Additional information

**How to cite this article:** Tsai, C. *et al*. Electrochemical generation of sulfur vacancies in the basal plane of MoS_2_ for hydrogen evolution. *Nat. Commun.* **8,** 15113 doi: 10.1038/ncomms15113 (2017).

**Publisher’s note:** Springer Nature remains neutral with regard to jurisdictional claims in published maps and institutional affiliations.

## Supplementary Material

Supplementary Information Supplementary Figures, Supplementary Tables, Supplementary Notes and Supplementary References
