# Supplementary material for: Electrochemical generation of sulfur vacancies in the basal plane of MoS2 for hydrogen evolution
Source: Nat Commun. 2017 Apr 21;8:15113. doi: 10.1038/ncomms15113 (PMC5530599; doi:10.1038/ncomms15113)
Supplement: Supplementary Information — Supplementary Figures, Supplementary Tables, Supplementary Notes and Supplementary References [file ncomms15113-s1.pdf]

Supplementary Table 1. Structures for MoS_2 with sulfur vacancies. The top-down view of the basal plane is shown. Only the lowest energy configurations of sulfur (S)-vacancies at each S-vacancy concentration are shown. Yellow atoms represent S and green atoms represent Mo.

S-vacancy Concentration			
0.0%	3.1%	6.2%	9.4%
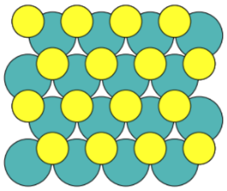	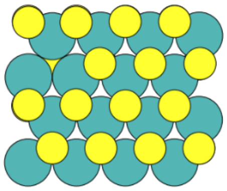	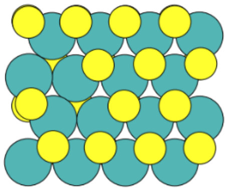	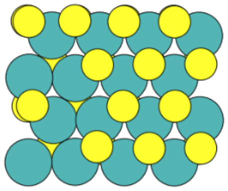
12.5%	15.6%	18.8%	21.9%
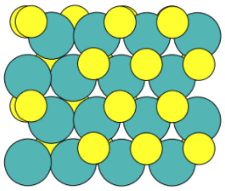	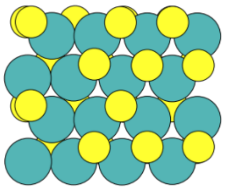	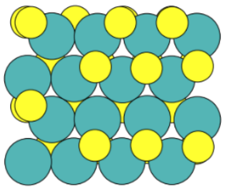	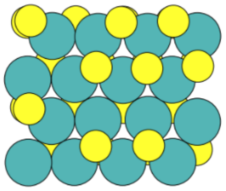

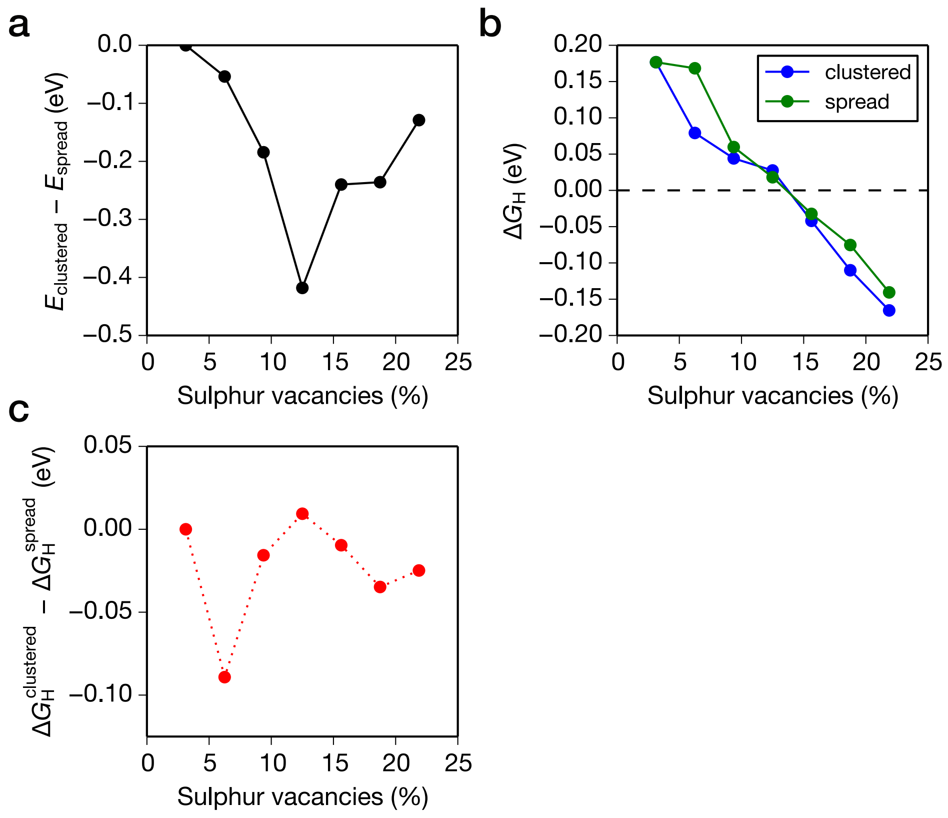

Supplementary Figure 1. Comparisons between clustered and spread out S-vacancies. (a) Change in stability of the total energy of MoS_2 with clustered S-vacancies compared to that with evenly spread out, as a function of the % of S-vacancies. (b) The hydrogen binding energy ΔG_H as a function of the % of S-vacancies for both clustered S-vacancies and evenly spread out vacancies. The optimal adsorption strength of $\Delta G_H = 0$ eV is shown as a dashed line. (c) Difference in ΔG_H between the clustered and evenly spread out S-vacancies at each % of S-vacancies.

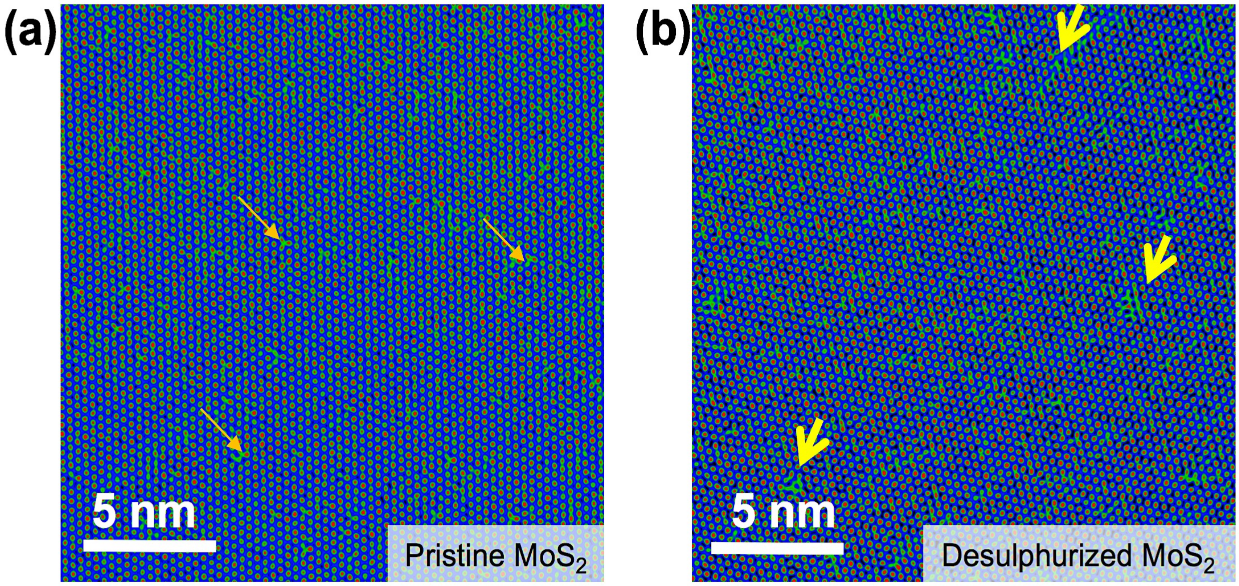

Supplementary Figure 2. False color TEM images obtained with a low electron-beam dose. The dose (10^3 electron/Å^{2}s dose rate) included a short exposure time (less than 30 s). (a) A pristine monolayer MoS_2 and (b) an electrochemically desulfurized monolayer MoS_2. The false color is applied to the image to make S-vacancies more clearly[4]. The red dots correspond to the empty areas between Mo and S atoms and the greenish color between the red dots represent the S-vacancies. The thin orange arrows indicate single vacancies and the thick yellow arrows label vacancy clusters.

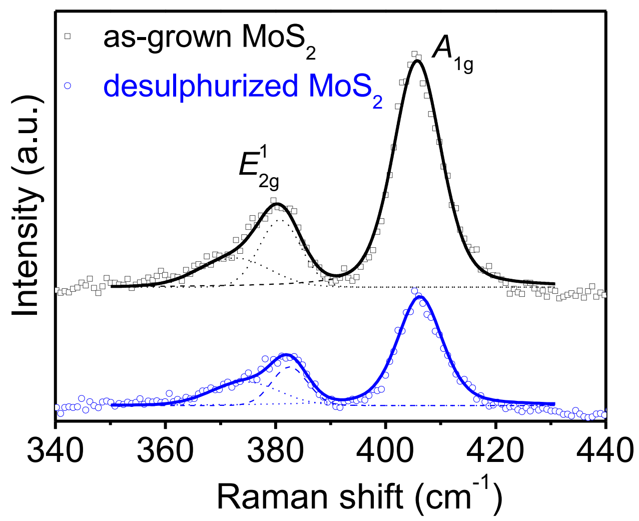

Supplementary Figure 3. Raman spectra for polycrystalline MoS_2 on carbon foam. Symbols represent measurement data and lines are fitting curves. Upper spectra (black) are for as-grown MoS_2 and lower spectra (blue) are for desulfurized MoS_2.

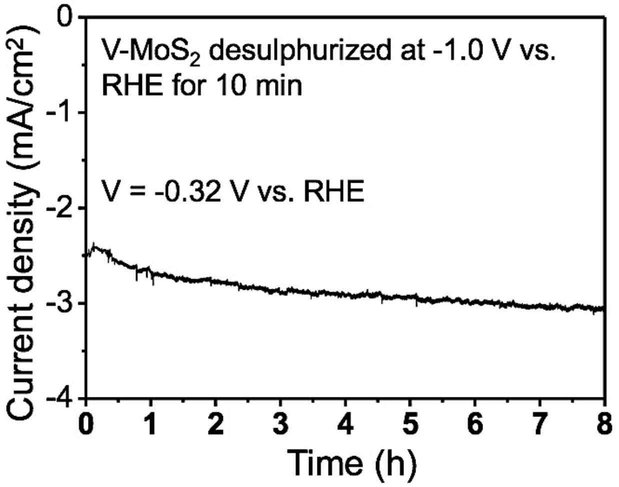

Supplementary Figure 4. Stability test for desulfurized polycrystalline multilayer MoS_2 on carbon foam. The sample was desulfurized at -1.0 V vs. RHE for 10 min prior to stability test that is conducted at constant potential of – 0.32 V vs. RHE for 8 h.

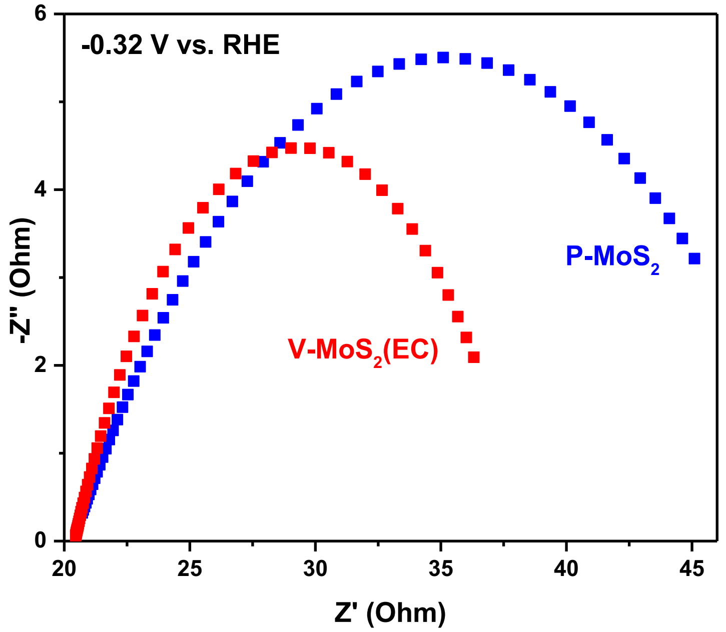

Supplementary Figure 5. Electrochemical impedance spectroscopy Nyquist plots. These were obtained for desulfurized polycrystalline multilayer MoS_2 on carbon foam at –0.32 V vs. RHE. Blue symbols are for as-grown MoS_2 and red symbols are for desulfurized MoS_2. The desulfurization potential and duration are –1.0 V vs. RHE and 10 min, respectively. R_s of pristine MoS_2 and desulfurized MoS_2 on carbon foam are 21.09 Ohm and 20.34 Ohm, respectively.

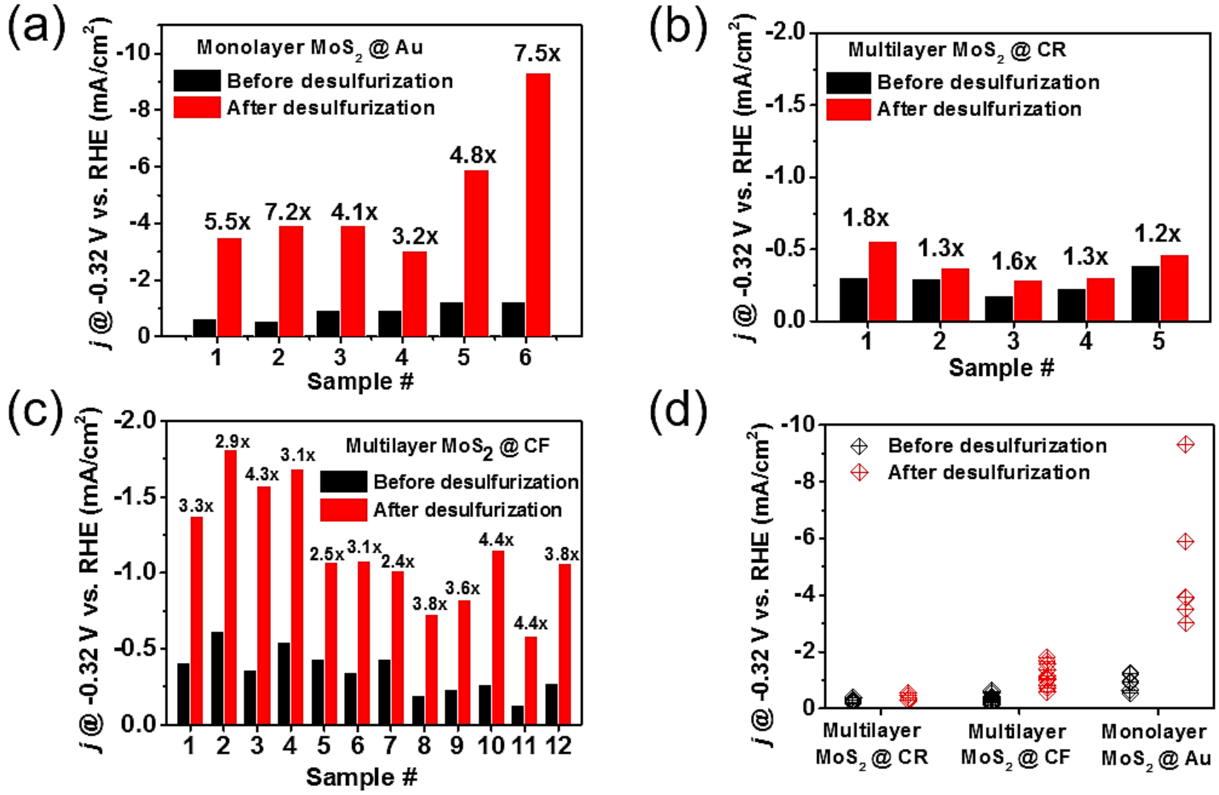

Supplementary Figure 6. Statistical data on the current density before (black bar) and after EC desulfurization process (red bar) for MoS_2 supported on different substrates. (a) Monolayer MoS_2 on Au substrates, (b) multilayer MoS_2 on carbon rod substrates, and (c) multilayer MoS_2 on carbon foam substrates. (d) Summary of all the samples in (a) to (c).

Supplementary Table 2. Comparison of potentials needed to reach a TOF of 2 H_2 atoms per second. Data is extracted from main Figure 5b. Our catalysts are labelled by *.

**MoS_x based HER catalysts**	***E* vs. RHE @ TOF = 2 H_2/s**
Edge-site only	– 0.120
Desulfurized monolayer MoS_2 on Au*	– 0.135
Edge and basal plane sites	– 0.145
$[Mo_3S_{13}]^{-2}$ | HOPG	– 0.185
iR-corrected desulfurized multilayer MoS_2 on carbon foam*	– 0.210
Double gyroid MoS_2	– 0.225
$[Mo_3\ S_{13}]^{-2}$ | graphite paper (GP)	– 0.225
Desulfurized multilayer MoS_2 on carbon foam*	– 0.245

*This work

Supplementary Note 1. Computational setup of the atomic structures with S-vacancies

As in our previous work[1], each computational unit cell consists of 16 Mo atoms (green circles) and 32 S atoms (yellow circles) on the surface, as shown in Supplementary Table 1. The concentration of S-vacancies is defined as (number of S-vacancies)/(number of total S atoms), which allows us to vary the S-vacancy concentration in 3.125% increments. The surface energies were determined relative to pristine monolayer MoS_2 (no S-vacancies) following the methodology from previous work[1]:

$$\gamma = E_{\mathrm{tot}} - N_{\mathrm{Mo}} E_{\mathrm{bulk}}^{\mathrm{MoS_2}} + (2N_{\mathrm{Mo}} - N_{\mathrm{S}})\,\mu_{\mathrm{S}} - N_{\mathrm{H}}\mu_{\mathrm{H}} \quad (1)$$

S-vacancies were found to form in clusters, because it is more exergonic to form a vacancy close to an existing one.

The effect of having clustered vacancies is thus only to make it easier to form certain S-vacancy concentrations. This was not considered in our previous work, where S-vacancies were evenly spread out to correspond to the results from Ar-plasma treatment. All stable structures for each S-vacancy concentration are shown Supplementary Table 1. Our results are in good agreement with calculations performed by Le et. al. [2], in that S-vacancies preferentially form in neighbouring sites. They find that the periodically repeating and continuous row of S-vacancies is the most stable, which we also find with the 12.5% S-vacancies in our unit cell. Although they reported a formation of a continuous row, we find that the zigzag structure is slightly more stable, by ~0.02 eV. This small difference could be due to differences in the employed exchange-correlation functional. Le et. al. also reported diffusion barriers for the S-vacancies, finding them to be insurmountable for most coverages. Hence, we have assumed that S-vacancies form successively without an intermediate step for reorganization.

Supplementary Note 2. Comparison between the clustered and spread out S-vacancies

Although S-vacancies were found to be more stable when formed in clusters rather than being evenly spread out, this is found not to affect the associated trends in hydrogen binding energy, and hence HER activity. Supplementary Figure 1 summarizes various comparisons between MoS_2 with clustered and evenly spread out S-vacancies. The additional stabilization in the surface energy due to clustering increases, and then decreases again (Supplementary Figure 1a) as the number of S-vacancies increase. Since there are few S-vacancies available for clustering at the small concentrations, the stabilization is limited. On the other hand, when there is a large concentration of S-vacancies, they are in neighboring sites even when being evenly spread out. The largest amount of stabilization is thus in the intermediate region. The reactivity of the sites is not strongly affected by the location of the S-vacancies (Supplementary Figure 1b), and the hydrogen binding strengths, and thus HER activity, are roughly unchanged throughout the range of S-vacancies. This is illustrated in Supplementary Figure 1c, where the maximum difference in ΔG_H is well within 0.1 eV.

Supplementary Note 3. TEM characterization of sulfur vacancies

The electrochemical desulfurization process was applied to create S-vacancies in the as-grown pristine monolayer MoS_2 transferred to a Au coated Si wafer. Then the desulfurized monolayer MoS_2 was transferred to a TEM grid using the PMMA-assisted transfer method, and then characterized with a FEI Titan environmental TEM 80-300 operated at 80kV. The TEM images of a pristine and desulfurized monolayer MoS_2 samples are shown in Supplementary Figure 2 for comparison, where a false color is applied to the image to make S-vacancies clearer. We confirmed that negligible sulfur vacancy can be created in monolayer MoS_2 under the low-dose electron beam with 10^3 electron/Å^{2}s dose rate for less than 30 s. Comparing Supplementary Figure 2a and Supplementary Figure 2b, one can see that (1) there are some intrinsic sulfur vacancies present in the pristine monolayer MoS_2 that could come from the growth or/and transfer processes; most of these sulfur vacancies exist as isolated individual vacancies, and (2) the desulfurized monolayer MoS_2 has a much higher density of sulfur vacancies and many of these vacancies form clusters. These TEM images suggest that electrochemical desulfurization has created additional sulfur vacancies.

Supplementary Note 4. Raman characterization of polycrystalline multilayer MoS_2 before and after desulfurization

The Raman spectra of polycrystalline multilayer MoS_2 grown on carbon foam substrate are shown in Supplementary Figure 3. Two peaks E^1_{2g} (in-plane mode) and A_{1g} (out-of-plane mode) are seen, suggesting the 2*H*-phase MoS_2. The broaden peaks indicate the polycrystalline multilayer nature of the MoS_2 film[3]. After desulfurization, the Raman peaks show negligible change, implying that 2*H*-phase is still dominant. It is worth noting that Raman collects signals from all layers, and thus the Raman spectrum of multilayer MoS_2 is not sensitive enough to probe the sulfur vacancy formation as that in monolayer MoS_2 [1].

Supplementary Note 5. Stability test for desulfurized MoS_2 on carbon foam

As stated in the manuscript, the durability of desulfurized multilayer MoS_2 supported on carbon foam allows us to study the tunability of HER activity. Supplementary Figure 4 shows an 8-h long stability test under -0.32 V vs. RHE for a sample desulfurized at –1.0 V vs. RHE for 10 min.

Supplementary Note 6. Electrochemical impedance spectroscopy for multilayer MoS_2 on carbon foam

The electrochemical impedance spectroscopy (EIS) Nyquist plots of polycrystalline multilayer MoS_2 grown on carbon foam substrate are shown in Supplementary Figure 5. The EIS measurements were constructed at the applied potential of –0.32 V vs. RHE from the high frequency of 200 kHz to the low frequency of 1 Hz in 0.5 M H_2SO_4 electrolyte solution. Z' is the real impedance and Z" is the imaginary impedance. The series resistance (R_s) of pristine MoS_2 and desulfurized MoS_2 on carbon foam were measured at the high frequency intercept as 21.09 Ohm and 20.34 Ohm, respectively, and used for iR correction. All the measured samples are summarized in Supplementary Figure 6. A quantitative comparison between our samples and state-of-the-art MoS_x HER catalyst is presented in Supplementary Table 1.

Supplementary References

1. Li, H. *et al.* Activating and optimizing MoS_2 basal planes for hydrogen evolution through the formation of strained sulfur vacancies. *Nat. Mater.* **15**, 48–53 (2016).
2. Le, D., Rawal, T. B. & Rahman, T. S. Single-Layer MoS_2 with Sulfur Vacancies: Structure and Catalytic Application. *J. Phys. Chem. C* **118,** 5346–5351 (2014).
3. Li, H. *et al.* From Bulk to Monolayer MoS_2: Evolution of Raman Scattering. *Adv. Funct. Mater.* **22,** 1385–1390 (2012).
4. Yu, Z. *et al*. Towards Intrinsic Charge Transport in Monolayer Molybdenum Disulfide by Defect and Interface Engineering. *Nat Commun.* **5**:5290 (2014) 10.1038/ncomms6290.
